# Revisiting behaviour of monometallic catalysts in chemical vapour deposition synthesis of single-walled carbon nanotubes

**DOI:** 10.1098/rsos.180345

**Published:** 2018-08-15

**Authors:** Rong Xiang, Shigeo Maruyama

**Affiliations:** 1 Department of Mechanical Engineering, The University of Tokyo, Tokyo 113-8656, Japan; 2 Energy NanoEngineering Laboratory, National Institute of Advanced Industrial Science and Technology (AIST), Tsukuba 305-8564, Japan

**Keywords:** single-walled carbon nanotube, catalyst, transmission electron microscopy

## Abstract

A catalyst is essential for the controlled synthesis of single-walled carbon nanotubes (SWNTs) by chemical vapour deposition (CVD). However, it is difficult to observe these nanosized particles in their original forms and in a statistical manner, which has resulted in a vague understanding of the behaviours of these particles. We present a technique to solve this long-standing issue. The key is to have an MEMS fabricated suspended SiO_2_ layer, which is thick enough to support catalyst deposition and nanotube growth but thin enough to allow electron beams to transit. On a 20 nm SiO_2_ film, we confirm that catalyst can be observed at an atomic resolution, and the catalyst–SWNT junctions can also be routinely observed. As a demonstration of this technique, we revisited the behaviour of monometallic catalysts through a systematic investigation of the size, chemical state and crystal structure of particles before and after high-temperature CVD. The active catalyst is found to follow a tangential growth mode, while the inactive catalyst is divided into three mechanisms: size growth, metal loss and inappropriate precipitation. The latter two mechanisms were not possible to observe by previous techniques.

## 1. Introduction

The single-walled carbon nanotube (SWNT), as a representative one-dimensional nanomaterial, has attracted much attention in the past decades due to the outstanding properties and potential applications. To synthesize this unique one-dimensional (1D) structure, a nanosized particle is usually needed. It serves as the nucleation site for carbon precipitation, and therefore is essential for the structure of the product [1].

Great efforts have been made on the exploration and optimization of the catalyst. In 1996, the first report on chemical vapour deposition (CVD) synthesis of SWNTs employed pre-formed Mo particles as catalyst [2]. Soon after, in 1998, transition metals (in particular, Fe, Co, Ni) were proposed and confirmed to be more efficient [3]. They quickly became the most-used active sites for laboratory- and industrial-scale SWNT production. Different from transition metals, novel metals (Au, Pt, Pd, etc.) were traditionally inactive, but researchers found in 2006 that they could be activated to produce clean SWNTs with decent efficiency [4]. In the following years, a semiconductor [5], oxide [6], and more recently intermetallic compound [7] and carbide [8], were introduced as a new class of catalyst: solid catalyst. One major feature of these catalysts is their capability of producing SWNTs with selective conductance or chirality [7–9].

Though great progress has been achieved so far, previous explorations on the catalysts were in most cases based on empirical studies, which resulted in a very vague understanding of the behaviours of these catalysts and the growth mechanism of SWNTs. One main reason for this is the absence of a technique to characterize nanosized catalytic particles in a highly reliable manner. Transmission electron microscopy (TEM) is the most powerful and straightforward for visualizing catalysts, but the conventional method only sees a very small area. Furthermore, the catalysts are usually not in their original forms, either sitting on a different support (e.g. carbon or Si_3_N_4_ grid) or in a very different environment (e.g. low growth pressure in an e-TEM condition).

**Figure 1.**
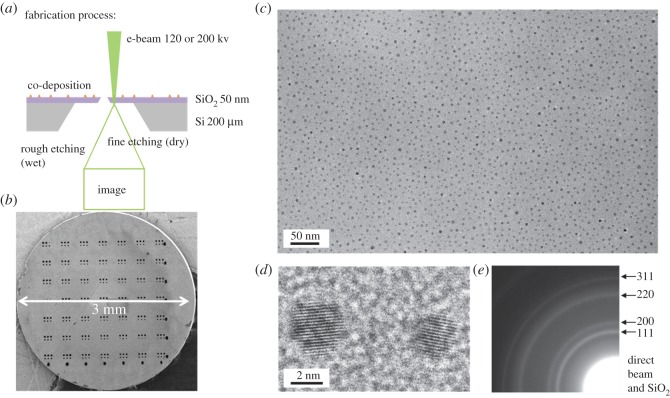
(*a*) A schematic of fabrication process of an MEMS fabricated Si/SiO_2_ TEM grid; (*b*) SEM image of the final 3 mm diameter product that fits a conventional TEM hold; representative (*c*) low-magnification and (*d*) high-magnification TEM images of Co nanoparticles sitting on a thin SiO_2_ film; (*e*) a selected area electron diffraction (SAED) pattern of Co catalyst obtained with an aperture diameter of 7 µm.

In this contribution, we present an attempt to address this two-decade-standing issue. The key we propose is to have an MEMS fabricated suspended SiO_2_ layer that is thick enough to support catalyst deposition and nanotube growth but thin enough to allow electron beams to transit (figure 1*a*). In this scenario, the catalyst particles are prepared and CVD is performed directly on this Si/SiO_2_ grid (figure 1*b*), similar to conventional processes on Si/SiO_2_ substrate. But the thin SiO_2_ region enables a transfer-free characterization of the catalyst/SWNTs and minimizes the information loss from CVD chamber to TEM column. Additionally, all images are taken from a bird's eye view, in which many particles are present simultaneously. We believe this study can provide so far the most statistical and intrinsic information of the nanosize particles used for CVD synthesis of SWNTs. As an example, monometallic catalysts are systematically re-investigated and their behaviours in CVD synthesis are revisited. New understanding on the catalyst structure, catalyst–SWNT junction and catalyst deactivation mechanism are illustrated.

## 2. Experimental set-up

**Figure 2.**
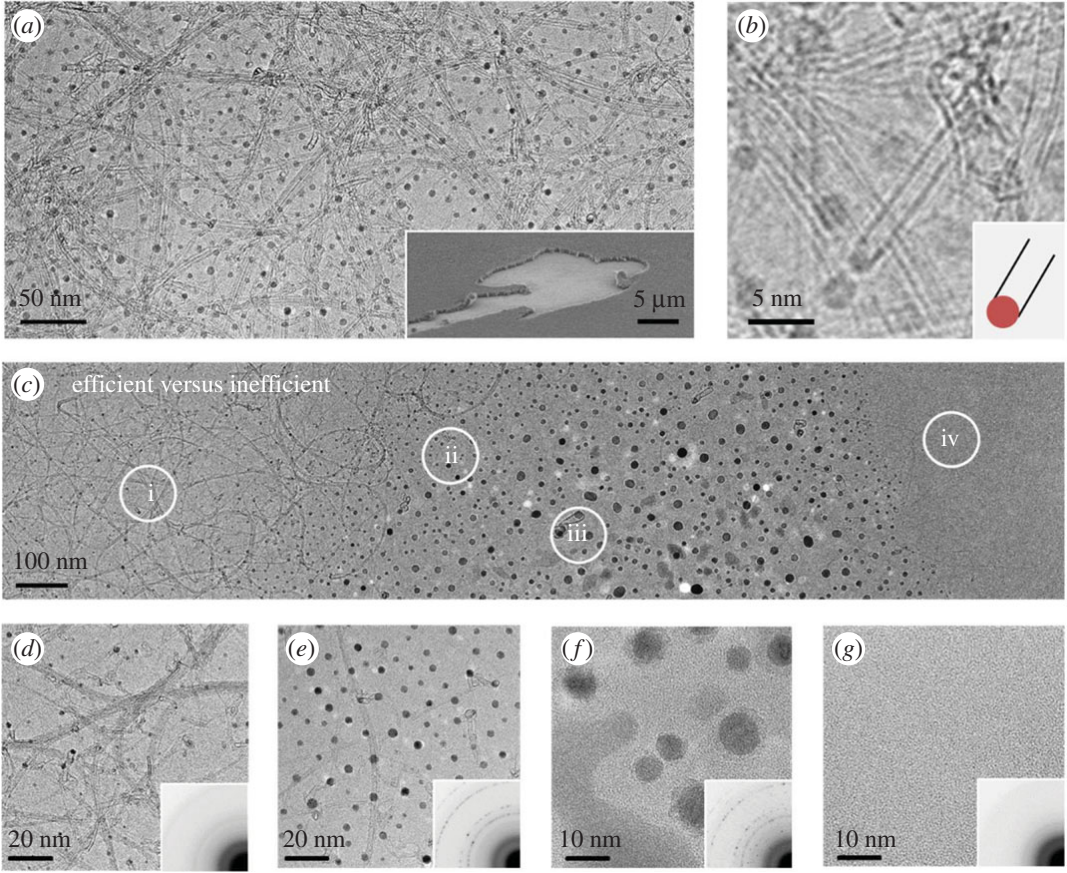
(*a*,*b*) Representative TEM images of catalyst particles on SiO_2_ grid after a short duration CVD: SWNTs are clearly observed in their original form, and the junction between a catalyst and a SWNT suggest a tangential growth mode; inset in (*a*) shows an SEM image of SWNTs grown on TEM grid. (*c*) TEM images of a region with gradient change of catalyst concentration, which reveal locally the difference between efficient and inefficient catalyst. (*d*)–(*g*) enlarged images and SAED patterns (inset) corresponding to the locations (i)–(iv).

Monometallic catalyst is prepared on the grid by sputtering. The nominal thickness is 0.3 nm in all cases. The catalyst in figure 2*c,d* is prepared by drop-casting a 50 µl catalyst solution (cobalt acetate in ethanol, 0.01 mmol l^−1^) directly on the grid. After catalyst deposition, the substrate is annealed in air at 400°C, followed by a reduction in a 40 kPa Ar diluted H_2_ (3%) atmosphere at 800°C for 10 min. After reduction, the grid is either taken out from the furnace for TEM characterization or SWNT growth is continued. SWNTs are grown in low-pressure alcohol catalytic CVD apparatus equipped with a dry rotatory pump [10]. The CVD temperature is 800°C for all cases in this study unless elsewhere stated, and pure ethanol is used as the carbon source without any carrier gas. Typically, after reduction, a flow of 450 sccm ethanol is introduced into the chamber at a pressure of 1.3 kPa. In order to avoid the overgrowth of SWNTs that are too thick for TEM observations, the ethanol flow is kept for only 2 s. However, because there is a delay in refreshing the chamber atmosphere, the total exposure time of catalyst to ethanol is around several seconds. After growth, ethanol feeding is stopped and the sample is cooled down to room temperature in a flow of 50 sccm Ar diluted H_2_ (3%). TEM images are obtained by using JEM-2000EX-II (JEOL Co. Ltd), JEM-2010F (JEOL Co., Ltd) and abberation corrected TEM ARM200 (JEOL Co., Ltd). All three TEMs are operated at 200 keV. Selected area electron diffraction (SAED) patterns are obtained by using JEM-2010F with a camera length of 60 cm at a parallel beam.

## 3. Results and discussion

### 3.1. Overview of imaging catalyst and single-walled carbon nanotubes on SiO_2_ film

Figure 1*a*,*b* shows the typical structure and a scanning electron microscope (SEM) image of the Si/SiO_2_ grid used in this study. The grid is fabricated by standard photolithography followed by wet and dry etching. A thin layer of SiO_2_ (20–50 nm) is used as the supporting film for the catalyst and serves as the observation window. This thin film and the whole grid is stable at high temperatures up to 1000°C. Therefore, after catalyst loading, the grid can be directly used for SWNT growth, which is similar to a standard Si/SiO_2_ substrate (electronic supplementary material, figure S1).

Figure 1*c* shows a representative low-magnification TEM image of monometallic Co catalyst sitting on a thin SiO_2_ film. The catalysts are prepared by sputtering and therefore have a good uniformity over the entire grid. Even seeing through the SiO_2_ film, one can clearly distinguish the strong contrast of nanoparticles from the amorphous background. The lattice fringe of the catalyst can also been routinely observed by a conventional TEM (figure 1*d*). The size of particles ranges from 2 to 6 nm, with a mean diameter of approximately 3 nm. This value is slightly large for growth of SWNTs, which is consistent with much previous empirical understanding that monometallic particles alone are usually not the most efficient. Some additional ingredients, e.g. Mo, Al, are needed to help disperse and prevent sintering of these catalysts at high temperatures [11,12]. This process will be demonstrated and discussed in detail in the next section.

As catalyst can be visualized in a large area, selective area electronic diffraction (SAED) is a powerful way to determine the crystal structure of the particles. The inset of figure 1*e* shows a typical SAED pattern of monometallic Co catalyst. Surprisingly, the pattern unambiguously suggests that these Co particles have a dominant facet centre cubic (fcc) structure, which is different from the classic phase diagram where Co is hexagonal close packed (hcp) at room temperature and transits to fcc only at a temperature above 700°C [13]. Though in contrast with almost all previous discussions (where Co was believed to be hcp) [14], this result is understandable because nanoparticles tend to form simple fcc structures at reduced size [15]. The density of these catalysts in this case is estimated to be 8000 µm^−2^, which is of the same order of magnitude as our previous measurement of a vertically aligned SWNT array [16]. This suggests our SAED pattern obtained from an area Φ 7 µm contains roughly 300 000 particles, meaning very statistical information of catalyst particles is obtained. This is a unique and powerful feature for catalyst investigations.

High-resolution images of the catalyst are consistent with the SAED assignment, where many particles show a lattice fringe of 2.0 Å, which corresponds to the (111) plane of fcc Co. Most particles present characteristic features as single crystals, but twin- and poly-crystals are also occasionally observed for larger particles. While most of the catalyst is confirmed to be fcc metallic Co, an oxide layer can be observed after the sample is exposed to air. In this case, overnight annealing in a reduction atmosphere, and/or introducing trace amounts of carbon at high temperatures help to keep the particles in metallic forms. One technical issue when obtaining high-resolution images on this special grid is that SiO_2_ is not conductive, which is a major difference from conventional grids made of Cu and carbon. On SiO_2_ films, it is critical to keep a low electron dose to obtain a stable image. With the reduced electron dose, the lattice fringe and atomic resolution can be repeatedly obtained.

This Si/SiO_2_ grid is stable at high temperatures so can be placed into a furnace for direct SWNT synthesis, as previously noted. Representative TEM images of the same grid after several seconds of CVD are shown in figure 2*a*. Single and bundled SWNTs are clearly observed on this SiO_2_ film in their as-grown form. The growth behaviour and growth rate on this TEM grid is similar to the case on standard Si/SiO_2_ substrate. After longer CVD, even thicker SWNTs can be achieved (SEM shown as inset of figure 2*a*). But significantly different from conventional substrate growth, SWNTs in this case can be directly characterized after CVD, no post-transfer or sampling processes are involved. If SWNTs are not too thick, catalyst can also be imaged. The enlarged image in figure 2*b* suggests the diameter of the SWNTs grown is almost the same as the catalyst particles. This confirms that SWNTs follow a tangential growth mode in this study [17]. However, this observation does not discount that monometallic catalyst may also follow a vertical mode, as CVD conditions and other parameters (e.g. carbon solubility) may affect the growth mechanism [18]. Though more efforts are required, studying the junction between a catalyst and SWNTs becomes possible by this approach.

When the catalyst is prepared with a gradient, the local difference between efficient and inefficient areas can be identified. Figure 2*c* shows one example of SWNTs grown from wet-prepared catalyst. The approximately 0.5 × 2 µm^2^ area can be clearly classified as four regions: (i) efficient, (ii) less efficient, (iii) not efficient and (iv) reference. Figure 2*d–g* shows the corresponding enlarged TEM images and SAED patterns of these four regions. Because the catalysts are subject to an exact CVD environment (temperature, pressure, even local flow), this difference in efficiency is solely attributed to the catalyst, that is, amount of metal precursor deposited in a local area. In region (i), small catalyst particles are formed and SWNTs are efficiently produced (figure 2*d*). However, in region (ii), where the amount slightly increases, most particles aggregate above 5 nm and the SWNT yield dramatically decreases (figure 2*e*). Large diameter SWNTs and few walled CNTs appear. In region (iii), where the metal amount further increases, both the image and SAED patterns reveal the coexistence of Co metal and Co oxide (figure 2*f*). This suggests the reduction is not even complete and explains the inefficiency. These differences were vaguely understood in previous batch-to-batch studies, but now can be clearly visualized by the current technique.

**Figure 3.**
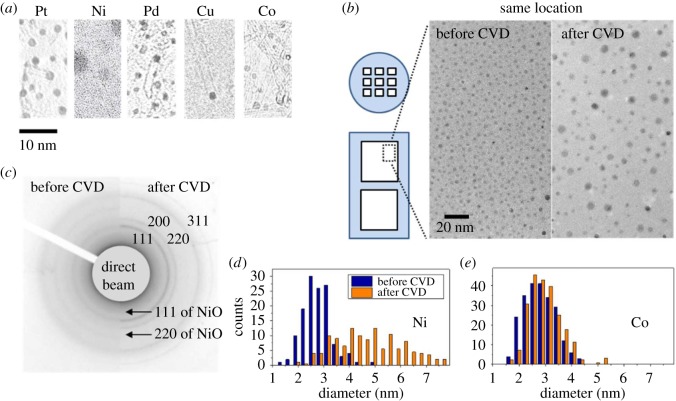
(*a*) TEM images of SWNTs synthesized from various monometallic particles sitting on an SiO_2_ film; (*b*) a comparison of the exact same location of an Ni catalyst before and after a high-temperature CVD; (*c*) corresponding SAED patterns of Ni catalyst; (*d*,*e*) diameter distribution histogram of (*d*) Ni and (*e*) Co catalyst on SiO_2_ before and after CVD, suggesting Ni deactivates due to the quick increase in particle size after carbon exposure.

With this technique, we systematically re-examined the size, density, chemical state and crystal structure of various monometallic catalysts before and after CVD, together with the yield, structure and growth mode of the produced SWNTs. The results are summarized and compared in table 1. Overall, SWNTs are observed in TEM for all metals except W, but the SWNT yield varies greatly from metal to metal (figure 3*a*). Only Co and Ni produce Raman detectable amounts of SWNTs, while other metals result in either short or long but very sparse SWNTs. This is probably why they are concluded to be ‘in-active’ or at least ‘inefficient’ after previous characterizations by, for example, Raman and/or SEM. However, the nature of the current technique allows access to much detailed information regarding the catalysts and an in-depth understanding of their behaviours. According to the observations, we classify these less efficient catalysts into three different mechanisms, discussed separately in the next section.

**Table 1.** Summary of behaviour of various monometallic catalysts before and after CVD. ⓞ, very high; ○, high; △, decent; ×, none.

	element	Co	Ni	Fe	Cu	Pt	Pd	W
bulk properties	structure	hcp	fcc	bcc	fcc	fcc	fcc	bcc
	m.p. (K)	1768	1728	1811	1357.77	2041.4	1828.05	3695
	b.p. (K)	3200	3003	3134	2835	4098	3236	6203
particle size	before CVD	2.6	2.8	6.0	4.6	2.3	2.3	2.9
	after CVD	2.8	5.1	7.7	5.9	2.4	2.7	n.a.
number density (μm^−2^)	before CVD	8×10^3^	1.2×10^4^	2×10^3^	4.2×10^3^	2.3×10^4^	1.8×10^4^	1.4×10^4^
	after CVD	3.8×10^3^	3.0×10^3^	2×10^2^	5.8×10^2^	2.0×10^4^	1.1×10^4^	n.a.
oxidation	before CVD	slight	medium	heavy	slight	metallic	metallic	metallic
	after CVD	metallic	metallic	metallic	metallic	metallic	metallic	n.a.
particle structure	before CVD	fcc	fcc	bcc	fcc	fcc	fcc	bcc
	after CVD	fcc	fcc	bcc	fcc	fcc	fcc	bcc
SWNT growth	yield	ⓞ	○	△	△	△	△	×
	diameter	1–3	2–5	2–5	2–5	approximately 1	1–2	n.a.
	length	long	long	long	medium	short	short	n.a.
	density	high	low	low	low	very high	very high	n.a.
	mode	tangential	tangential	vertical	both	tangential	tangential	n.a.

### 3.2. Three different mechanisms for inefficient catalysts

Because the SiO_2_ grid can be put into a furnace for SWNT growth and then taken out for a TEM re-check, imaging the exact same location before and after CVD becomes possible. One example is shown in figure 3*b*, comparing the morphology of Ni catalyst before and after a short time (few seconds) of CVD (electronic supplementary material, figure S2). It is clear from the images that an increase in particle diameter occurs after introducing the carbon source (ethanol). The original average particle size was less than 3 nm, but quickly increased to around 5 nm after the exposure to ethanol for a few seconds. Though a small number of 2–3 nm particles still remain, the overall efficiency drastically deteriorates. This explains the observations that Ni alone produces long but very sparse SWNTs. As a comparison, Co, the most efficient catalyst in this study, maintained its size after CVD, and yields much denser SWNTs. Also, the SAED patterns in figure 3*c* suggest Ni particles were partially oxidized before CVD but became pure metal after CVD. This also reveals that ethanol CVD is a typical reduction process, even though ethanol is an oxygen-containing molecule. For a comparison, the histograms of the particle size distribution of Ni and Co are shown in figure 3*d*,*e*, showing Co is able to maintain its size while Ni undergoes a clear particle coarsening.

However, we claim the size increase in Ni is carbon-induced. In a control experiment, we kept Ni in a carbon-free reductive atmosphere for more than 60 min (much longer than the time scale of a typical CVD process). After this long annealing time, the particles are still of a similar size to as-reduced catalyst (figure 3*b*), which clearly indicates that the size change for Ni is dominantly caused by carbon induction. This behaviour of Ni is also very different from Fe (electronic supplementary material, figures S3 and S4). In the case of Fe, most particles were already of an inappropriate size (about 5–6 nm shown in table 1) before CVD, therefore are intrinsically too large for an efficient growth of SWNTs. In this sense, we emphasize that for a monometallic catalyst, Ni is one promising candidate for high-efficiency synthesis, as long as the carbon-induced size increase can be refrained, e.g. by lowering the carbon feeding speed, i.e. the partial pressure of the carbon source in the chamber.

**Figure 4.**
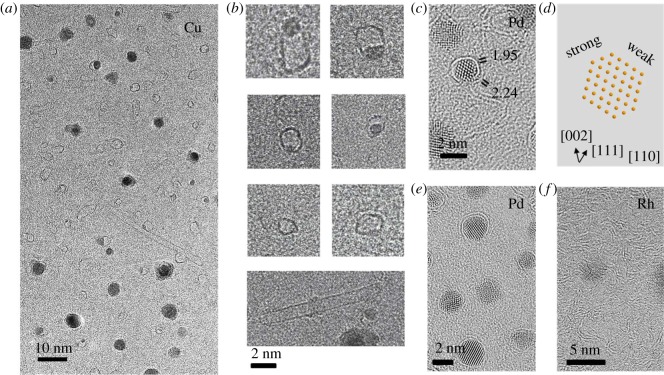
(*a*) A representative TEM image of Cu catalyst after CVD, showing many hollow mono-layer carbon spheres and SWNTs; (*b*) enlarged TEM images reveal that some spheres and SWNTs are connected with a particle but many others are metal-free; (*c*) a representative TEM image and (*d*) corresponding crystal structure of Pd catalyst, clearly suggesting a facet-dependent bonding of graphene layer; (*e*) a TEM image showing bilayer graphene coating occurs on many Pd particles; (*f*) a representative image of Rh particles, suggesting graphite layers precipitate everywhere and cover the whole substrate.

One interesting phenomenon was observed for the case of Cu, in which the deactivation (inefficiency) is due to metal loss. Cu is well known for its efficient production of mono-layer graphene but is under debate as a catalyst for SWNT growth [19]. While in most cases Cu is not regarded as an efficient catalyst, some growth of very long SWNTs from Cu catalyst are also reported [20]. In our observations, one characteristic feature for Cu is that many partial or completely hollow carbon spheres/nanotubes are observed (figure 4*a*). This phenomenon is different from all other metals investigated in this study. For Cu, there is little increase in particle size, but the particle density decreases by one order of magnitude after CVD (electronic supplementary material, figure S5). Even considering the diameter increase for individual particle, this result suggests a metal loss for Cu during the high-temperature CVD. Figure 4*b* shows some enlarged images for such spheres and SWNTs. Some SWNTs are clearly observed but found to be closed at both ends.

The mechanism for the formation of such hollow SWNTs is mysterious, but it is highly possible that the gradual loss of metal plays a dominant role. Previous calculations emphasized the importance of strong carbon–metal adhesion, and suggested catalyst may detach from a growing SWNT and generate hollow SWNTs [21]. However, from the tendency shown in figure 4*b*, some catalytic particles have similar diameter to the spheres or short SWNTs attached, while many other particles are smaller than the size of the carbon structure. These particles are possibly the intermediate stage for the metal-losing particles. If being subject to CVD for a longer time, the particles may further shrink and end up in a fully hollow structure. Therefore, it is almost certain that gradual metal loss is responsible for the formation of hollow spheres and SWNTs. We believe the driving of such metal loss for Cu is thermal evaporation, which is also routinely observed in the synthesis of graphene [22]. Though there is a possibility that metal atoms diffuse into the supporting SiO_2_ film, evaporation is more likely after considering the relatively low boiling temperature of Cu compared with other metals in this study. Therefore, we claim evaporation-induced metal loss is a second mechanism that can directly be observed by the current technique.

The third mechanism we demonstrate is inappropriate precipitation, which was observed for Pt, Pd and Rh. Different from the previous two cases in which catalyst particles expand (e.g. Ni) or shrink (Cu), these novel particles are stable, maintaining their size even in a high-temperature environment and after carbon deposition (electronic supplementary material, figure S6). The average particle size for Pt and Pd are about 2 nm and remain unchanged after CVD. Also, the particles dispersed on the SiO_2_ film at a much higher density (over 10^4^ per µm^2^) than other metals. However, efficient growth SWNTs is not achieved on these metals. As shown in figure 4*c*,*d*, in all cases, one to two layers of graphene can be observed on these metal particles, suggesting decomposition of the carbon source into solid carbon efficiently occurs. But difficulties in the precipitation of these carbon atoms into a tubular structure exist and seem to be responsible for the inefficiency. The mechanism slightly differs for Rh and Pd. For the former, many random carbon flakes are produced from an Rh particle, suggesting that the interaction between graphene layers and Rh metals could be very weak. This results in a random and isotropic precipitation of carbon in all directions, as shown in figure 4*f*.

In the latter case, however, the graphene seems to have a much stronger interaction with the metal surface, which makes it difficult for carbon atoms to precipitate into an SWNT. Figure 4*c* is a representative atomic resolution image of a 2 nm Pd particle viewing from the (110) direction. Clearly, graphene–metal interspacing on different facets can be distinguished. The facet-dependent growth of SWNTs has been discussed for a long time [23,24], but unfortunately, there has been very limited experimental evidence [25–27], particularly on SWNTs. In our case, the image clearly reveals the difference among facets. As shown in figure 4*c*, the carbon atoms on (002) are more firmly attached to Pd, while on (111) or (1–11) the carbon atoms are further from the surface (atomic structural scheme shown in figure 4*d*). But even on (111), the atoms only lift for a few angstroms up from the surface and fail to form an efficient cap for a continuous growth of SWNTs. From this observation, Pd's problem lies in its inefficient precipitation of tubular carbon even on the low energy (111) facet, which could possibly be due to the too strong bonding between carbon and Pd. Similar phenomena were observed for Pt, which produces only short SWNTs. If somehow activated, Pt and Pd may be effective for producing small-diameter and high-density SWNTs arrays, as their number density is highest among all the metals investigated in this study. In addition, Pd shows the potential to produce double-walled CNTs, as many Pd particles are wrapped with two layers of graphene. We did not present elemental analysis in this report as all catalysts are monometallic, but energy-dispersive X-ray spectroscopy and electron energy loss spectroscopy also work on our grid. Therefore, this technique is also applicable to bimetallic and other more complicated catalysts [28].

## 4. Conclusion

We presented a TEM approach that metal catalyst deposition and CVD synthesis of SWNTs can be performed on an MEMS fabricated thin SiO_2_ film. Nanosized particles and SWNTs grown from these particles can be imaged in their original morphology and in a statistical manner. Monometallic catalysts are used as examples. Their behaviours before and after CVD are systematically re-visited by this new technique. Monometallic Co produces SWNTs efficiently following a tangential growth mode, while other metals are classified into three different deactivation/inefficiency mechanisms: carbon-induced size growth, evaporation-driven metal loss and inappropriate precipitation. The first one is vaguely understood but has not been visualized before, and the latter two mechanisms were not possible to observe by previous techniques. Though some additional techniques are needed to support/explain the phenomenon, we believe the strategy proposed here serves as a key to the complicated catalytic synthesis of SWNTs. This method is also extendable to bimetallic or more complicated particles, catalyst behaviours at high temperatures or even other nanoscale 1D and 2D materials.

## Supplementary Material

Additional SEM and TEM images.

## Data accessibility

This article has no additional data.
