# Supplementary material for: Revisiting behaviour of monometallic catalysts in chemical vapour deposition synthesis of single-walled carbon nanotubes
Source: R Soc Open Sci. 2018 Aug 15;5(8):180345. doi: 10.1098/rsos.180345 (PMC6124116; doi:10.1098/rsos.180345)
Supplement: Additional SEM and TEM images. [file rsos180345supp1.pdf]

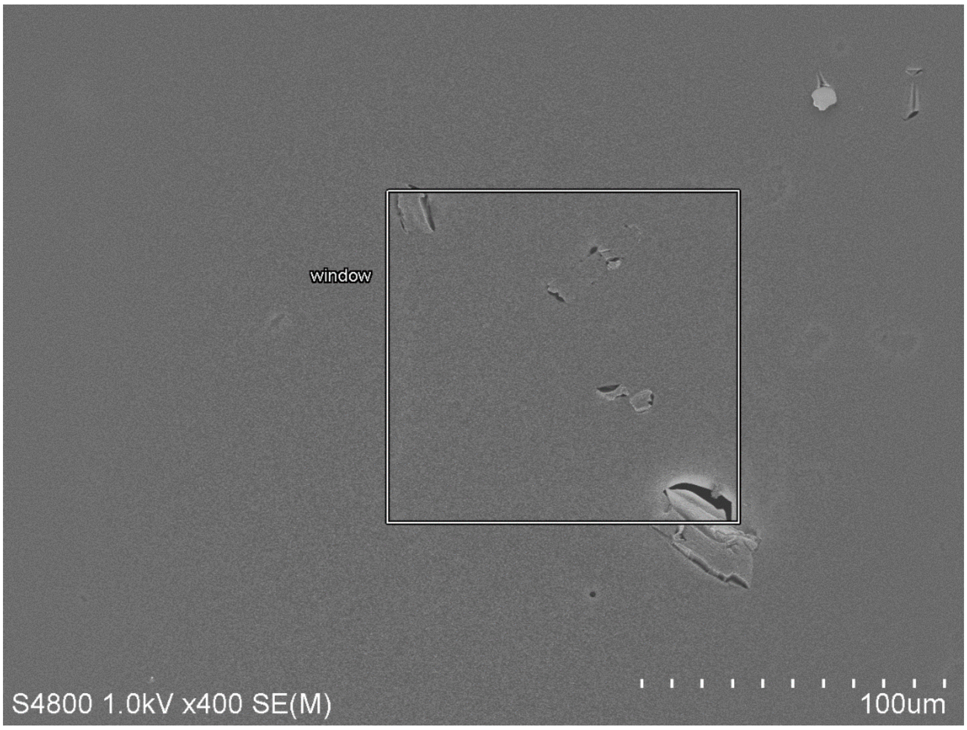

Figure S1 Original SEM image of SWNTs grown from Co catalyst on TEM grid (5 min growth), suggesting SWNTs grow efficiently and similarly on suspended SiO2 (window) region as the substrate region.

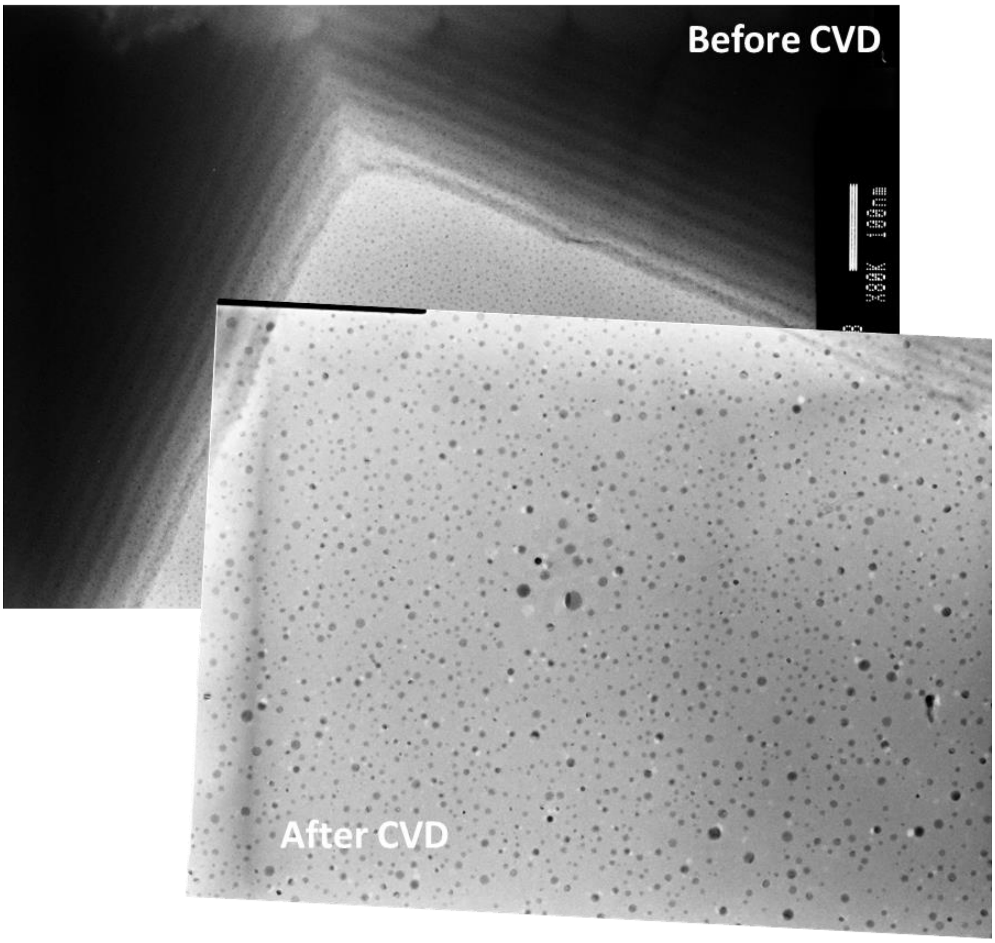

Figure S2 Original TEM images of Ni catalyst before and after growth, showing how images from the same location are obtained. To ensure a large viewing area, images are taken using film rather than CCD camera. Scans of the negative film are presented.

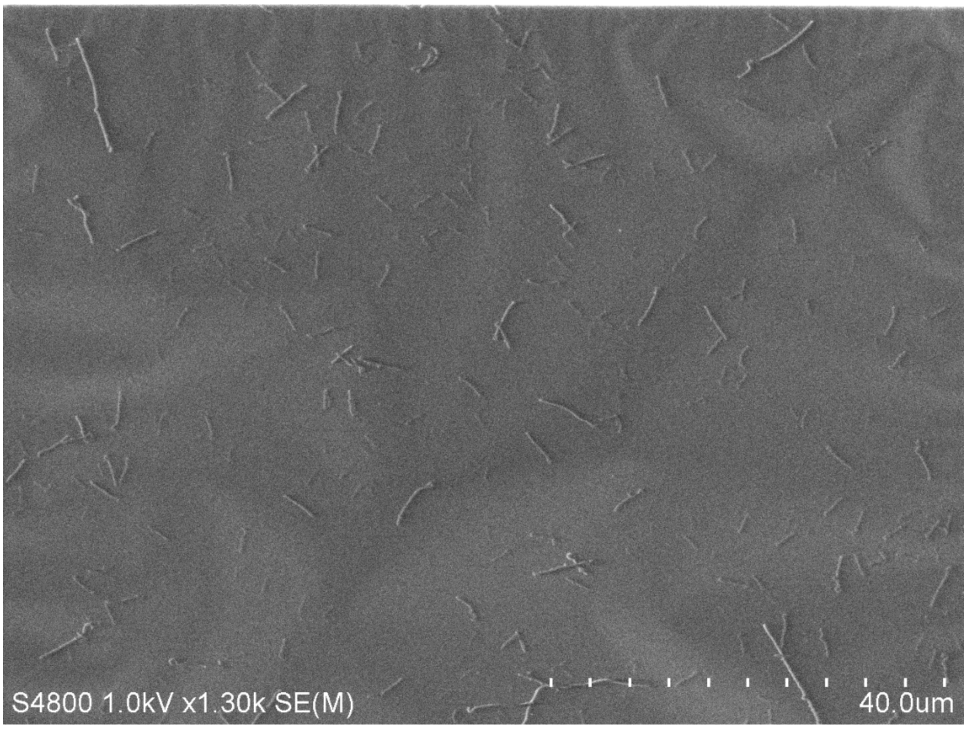

Figure S3 Original SEM image of SWNTs grown from Fe catalyst on suspended SiO2, showing long but only very sparse SWNTs can grow from monometallic Fe catalyst.

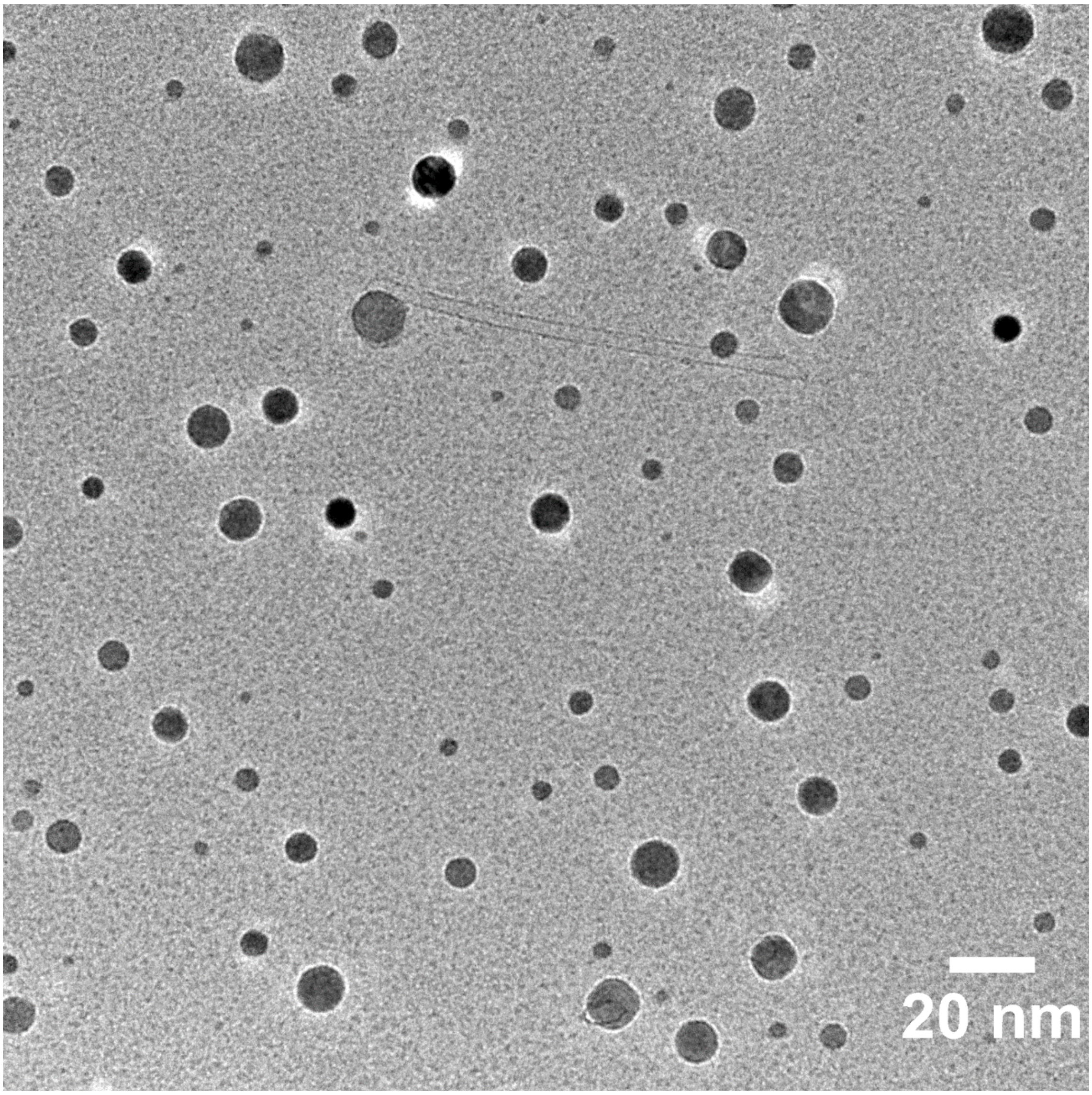

Figure S4 Original TEM image of SWNTs grown from Fe catalyst on suspended SiO2, showing the growth density is very small and SWNT diameter is smaller than catalyst, i.e., SWNTs follow a vertical growth mode.

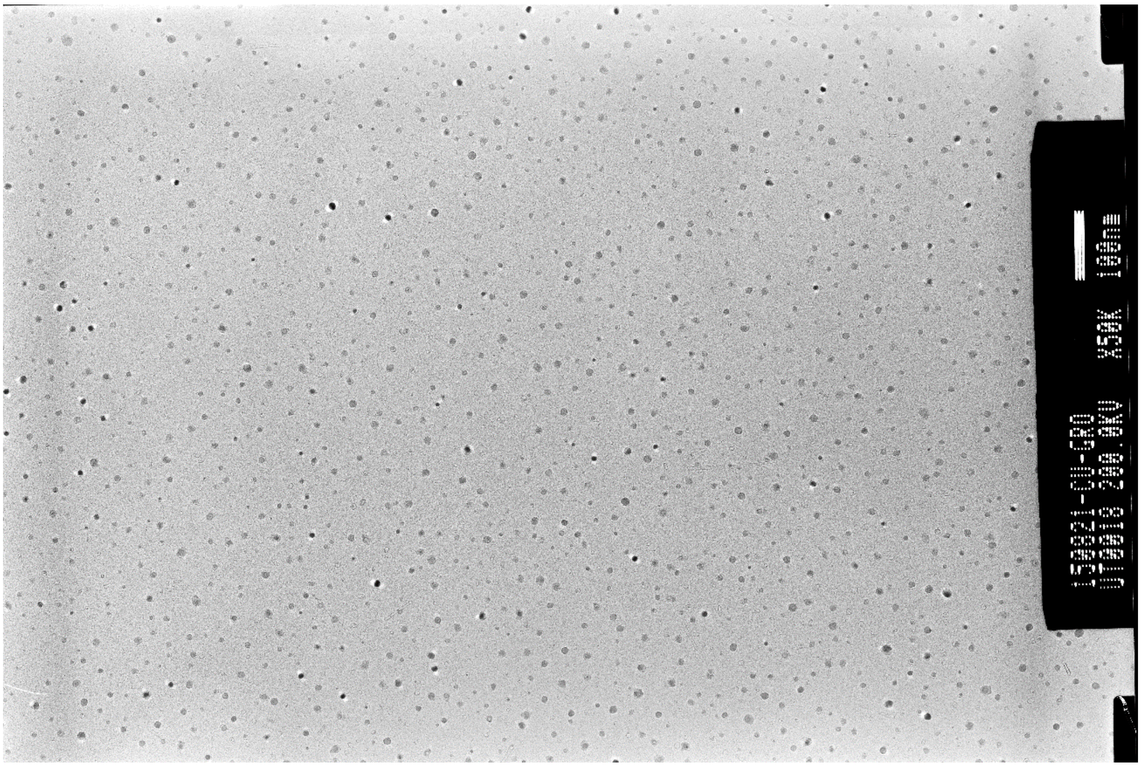

Figure S5 Original TEM images of Cu catalyst after SWNT growth, showing a very low catalyst density. Many hollow spheres and SWNTs can be uniformly observed on this grid. To ensure a large viewing area, images are taken using film rather than CCD camera. Scans of the negative film are presented.

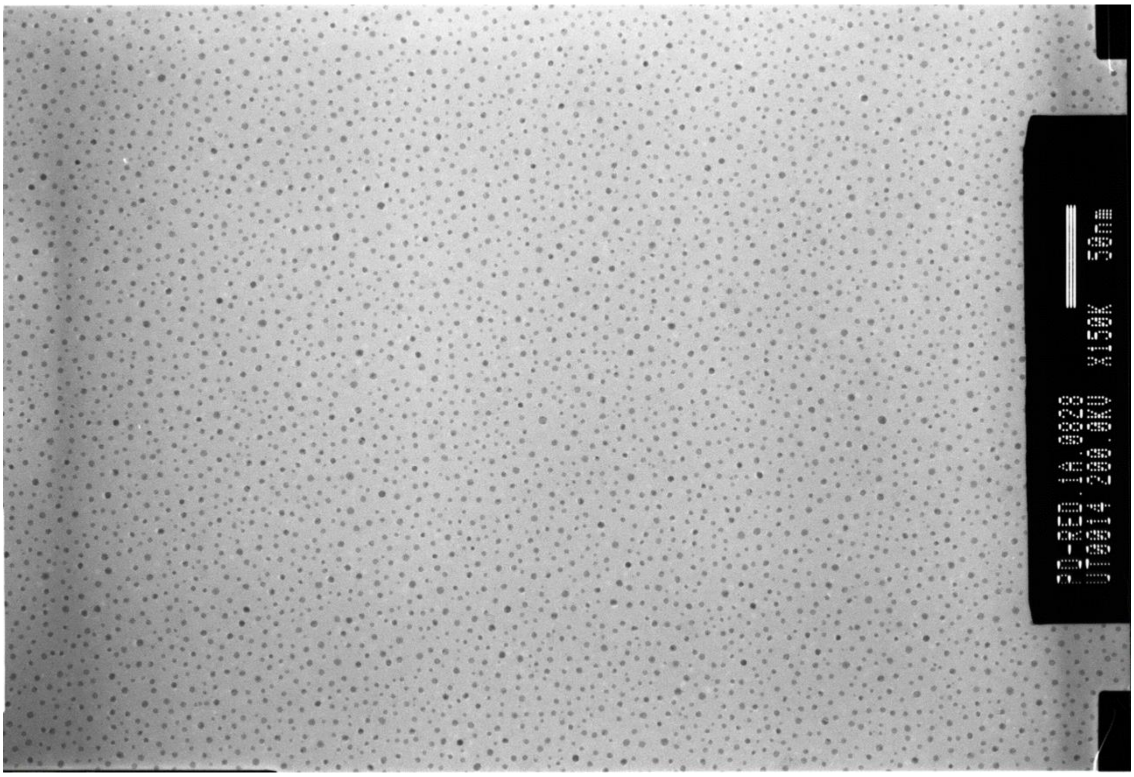

Figure S6 Original TEM images of Pd catalyst after reduction, showing a very high catalyst density. In comparison, the catalyst amount in Figure S5 is much less, which suggests that there is a significant loss of metal in case of Cu. To ensure a large viewing area, images are taken using film rather than CCD camera. Scans of the negative film are presented. The catalyst is extremely uniform on the entire grid.
